# Supplementary material for: A Role for Inositol Pyrophosphates in the Metabolic Adaptations to Low Phosphate in Arabidopsis
Source: Metabolites. 2021 Sep 4;11(9):601. doi: 10.3390/metabo11090601 (PMC8469675; doi:10.3390/metabo11090601)
Supplement: Supplementary file 1 [file metabolites-11-00601-s001.zip › TableS1.pdf]

Supplementary Table S1: Comparison of *vip* double mutants presented in this work, and others.

**Double mutant**	**At3G01310 (VIP1, VIH2)**<br>**T-DNA Insertion**	**At5G15070 (VIP2, VIH1)**<br>**T-DNA Insertion**	**Reference**
vip1-1/vip2-1	GK_204E06	SALK_094780	This work
vip1-1/vip2-2	GK_204E06	SAIL_175_H09	This work
vip1-2/vip2-2	GK_008H11	SAIL_175_H09	This work
vih1-2/vih2-4	GK_080A07	SALK_094780	Zhu et al. 2019
vih1-6/vih2-4	GK_080A07	SAIL_175_H09	Zhu et al. 2019
vih1-2/vih2-3	SAIL_165_H12	SALK_094780	Dong et al. 2019
vih1-3/vih2-3	SAIL_165_H12	SAIL_319_H07	Dong et al. 2019
