# Supplementary material for: A Role for Inositol Pyrophosphates in the Metabolic Adaptations to Low Phosphate in Arabidopsis
Source: Metabolites. 2021 Sep 4;11(9):601. doi: 10.3390/metabo11090601 (PMC8469675; doi:10.3390/metabo11090601)
Supplement: Supplementary file 1 [file metabolites-11-00601-s001.zip › TableS4.pdf]

Supplementary Table S4: Oligonucleotide primers used in this study.

Primer	Sequence
VIP1 F	5′ CAAGAAGTAGGTTCTTGTTTAAGCTTGGAGAC 3′
VIP1 R	5′ GTTGTGGAAGAGGTTTACGAGTCGCTC 3′
VIP2 F	5′ CAAGAGGTTGGTTCATGTTTGACCCTC 3′
VIP2 R	5′ GTGGAAGGGCCAAAGTTTAACAAGACG 3′
PP2A F	5′ TAACGTGGCCAAAATGATGC 3′
PP2A R	5′ GTTCTCCACAACCGCTTGGT 3′
PEX4 F	5′ CTTAACTGCGACTCAGGGAATCTTCTAAG 3′
PEX4 R	5′ TCATCCTTTCTTAGGCATAGCGGC 3′
PLDZ2 F	5′ TCACGACAAGCAAGAACAGGTTAG 3′
PLDZ2 R	5′ AGTGCAGAGGAAGAGCACCATC 3′
SPX1 F1	5′ GATTCCATTGTTGGAGCAAGA 3′
SPX1 R1	5′ AATCTGTTAGCTTCTTCTATTGTA 3′
GDPD1 F	5′ TGATGCAGCCCTTCTTGTCAGG 3′
GDPD1 R	5′ CTCCGTTGGTCAAGAAGAATACGG 3′
MGD2 F	5′ ATGGATGGGAGCTTGTGACTGC 3′
MGD2 R	5′ GGCACATTCCCTTTCTCCTGTC 3′
MGD3 F	5′ ACACTTGCCACCGTACATGGTTC 3′
MGD3 R	5′ TGCTCTCTTTGCAACCTCTTTGG 3′
PHT1;5 F	5′ CGCTCTTGTAGCTCGGAATAC 3′
PHT1;5 R	5′ TCCACTTGAAGCACCTTAGACA 3′
NPC4 F	5′ GGATTGAGCCTGGCACAGTT 3′
NPC4 R	5′ GACGAATGCTCATATTGTGACCTT 3′
PS2 F	5′ TCTTGAGAACAATCCCAATTCATC 3′
PS2 R	5′ CCTAAGCTCACACCCTAAATCATGT 3′
SQD2 F	5′ AAC CGG TTC TGT GTG CAA GCT G 3′
SQD2 R	5′ AGC CTC TCG TCT GAC CAC CTT TAC 3′

AT4 F	5′ AGG AAC ACA CCT GAA TGG TGC 3′
AT4 R	5′ CCG TGT TTA CTT TGT TTC CGG T 3′
IPS1 F	5′ TTTGGAGAATAGTCAGACCAGTGC 3′
IPS1 R	5′ TCACTATAAAGAGAATCGGAAGCA 3′
