# Supplementary figures and images for: A Role for Inositol Pyrophosphates in the Metabolic Adaptations to Low Phosphate in Arabidopsis
Source: Metabolites. 2021 Sep 4;11(9):601. doi: 10.3390/metabo11090601 (PMC8469675; doi:10.3390/metabo11090601)

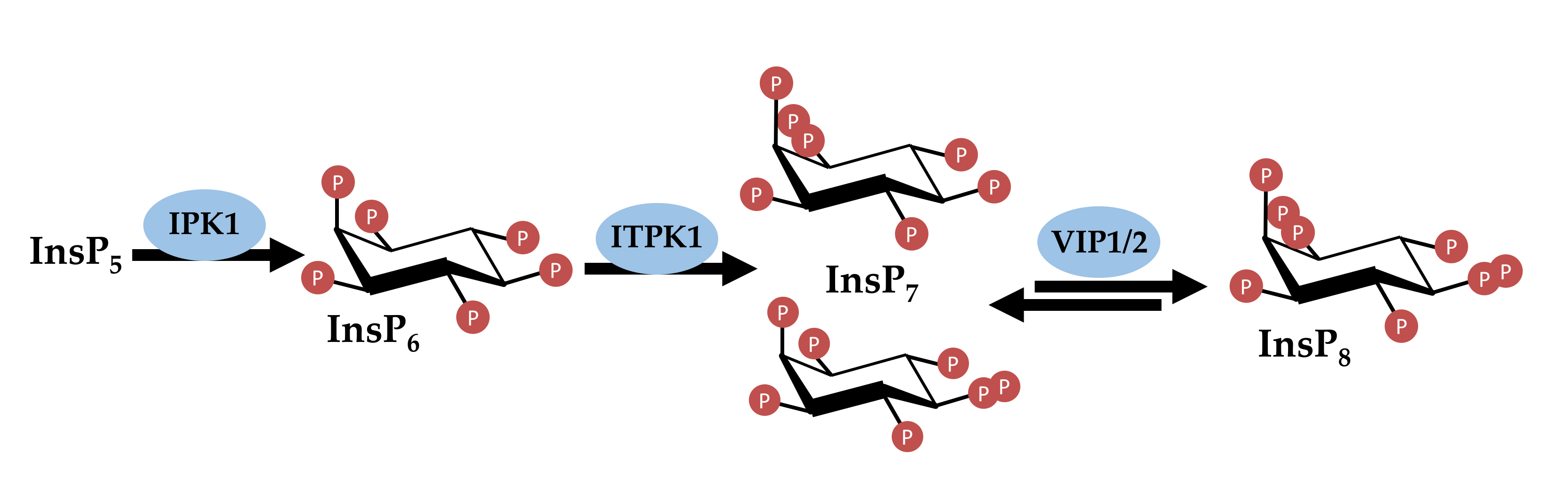

Supplement: Supplementary file 1 [file metabolites-11-00601-s001.zip › FigureS1.tiff]

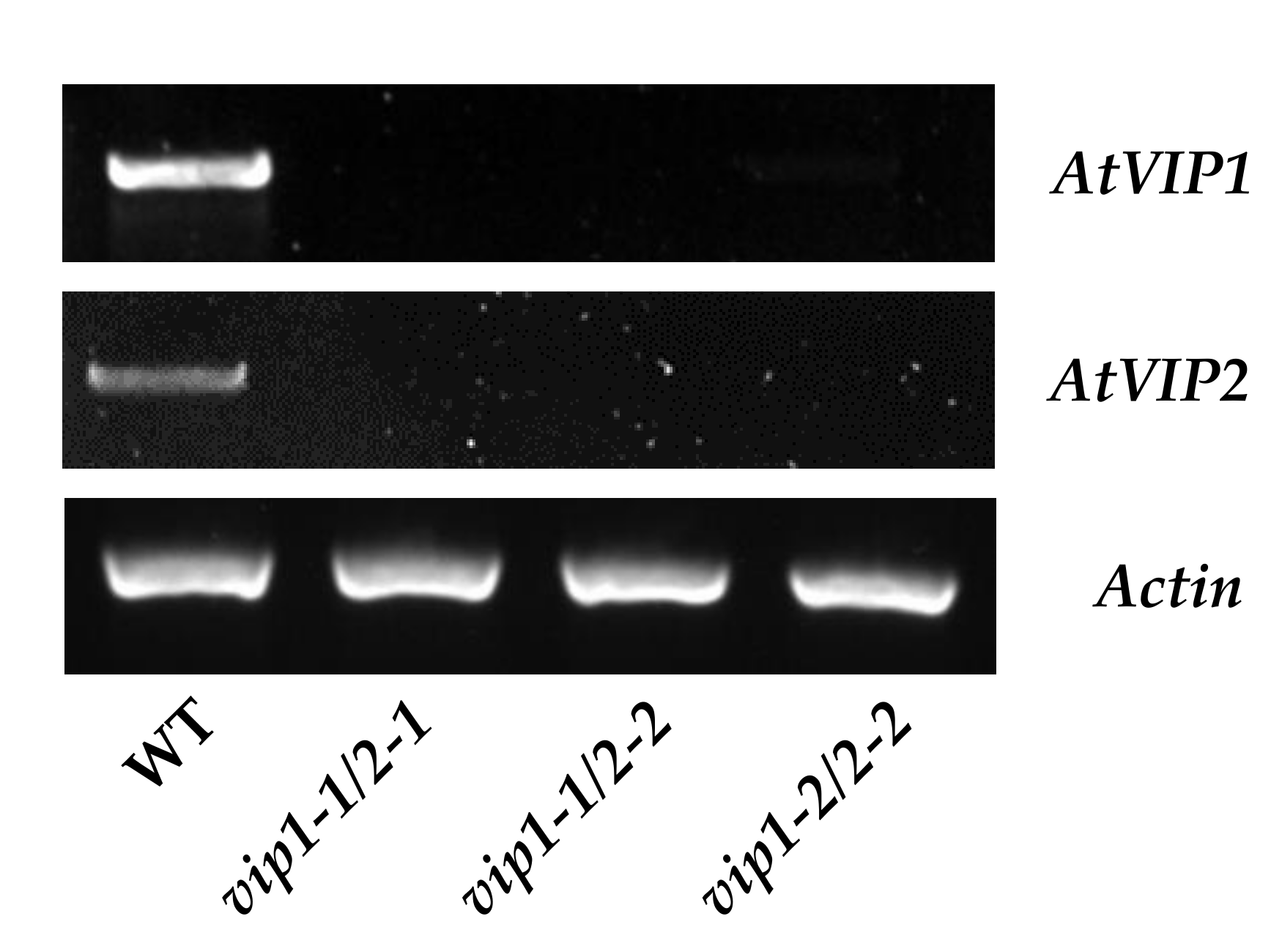

Supplement: Supplementary file 1 [file metabolites-11-00601-s001.zip › FigureS2.tiff]

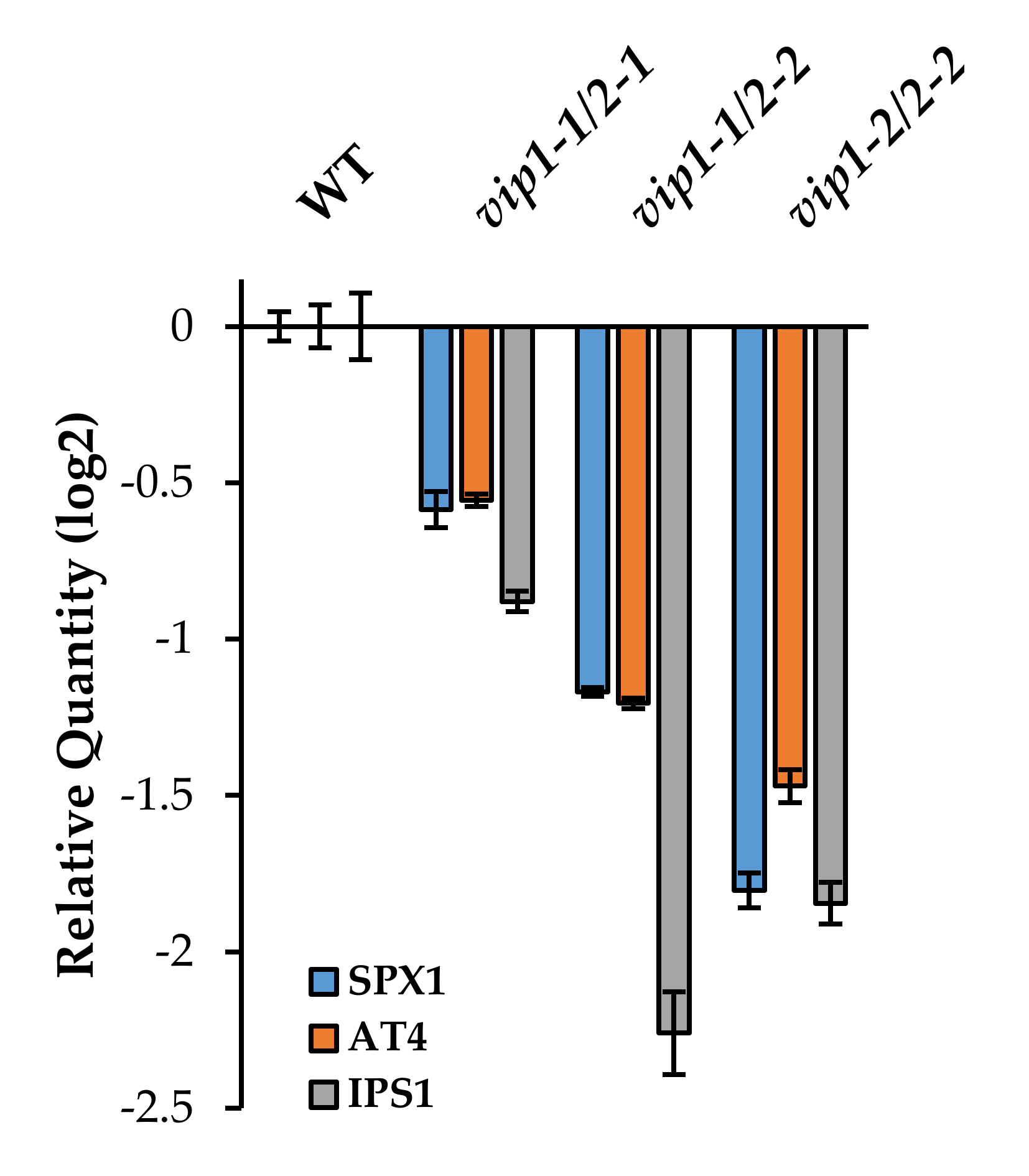

Supplement: Supplementary file 1 [file metabolites-11-00601-s001.zip › FigureS3.tiff]

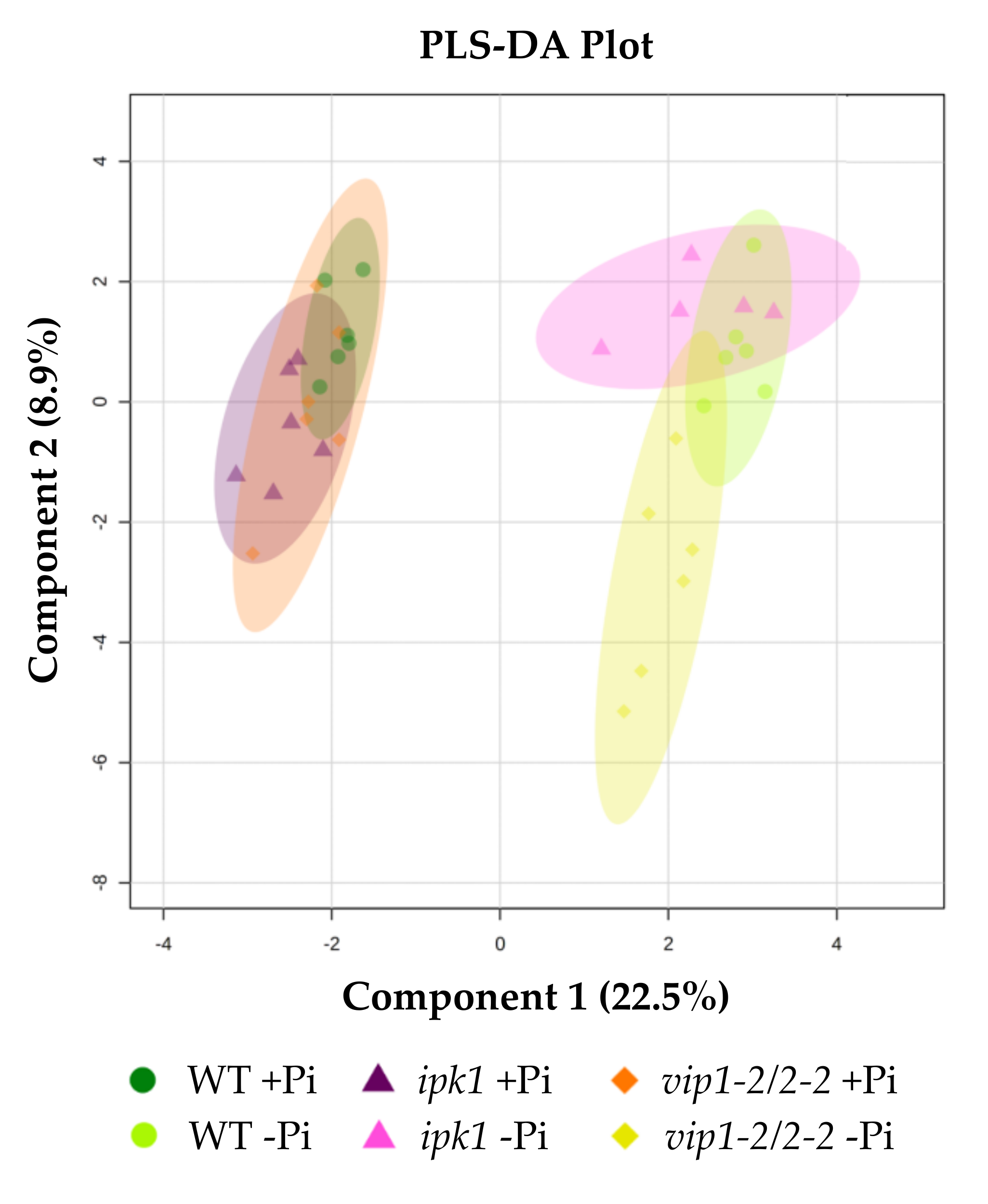

Supplement: Supplementary file 1 [file metabolites-11-00601-s001.zip › FigureS4.tiff]

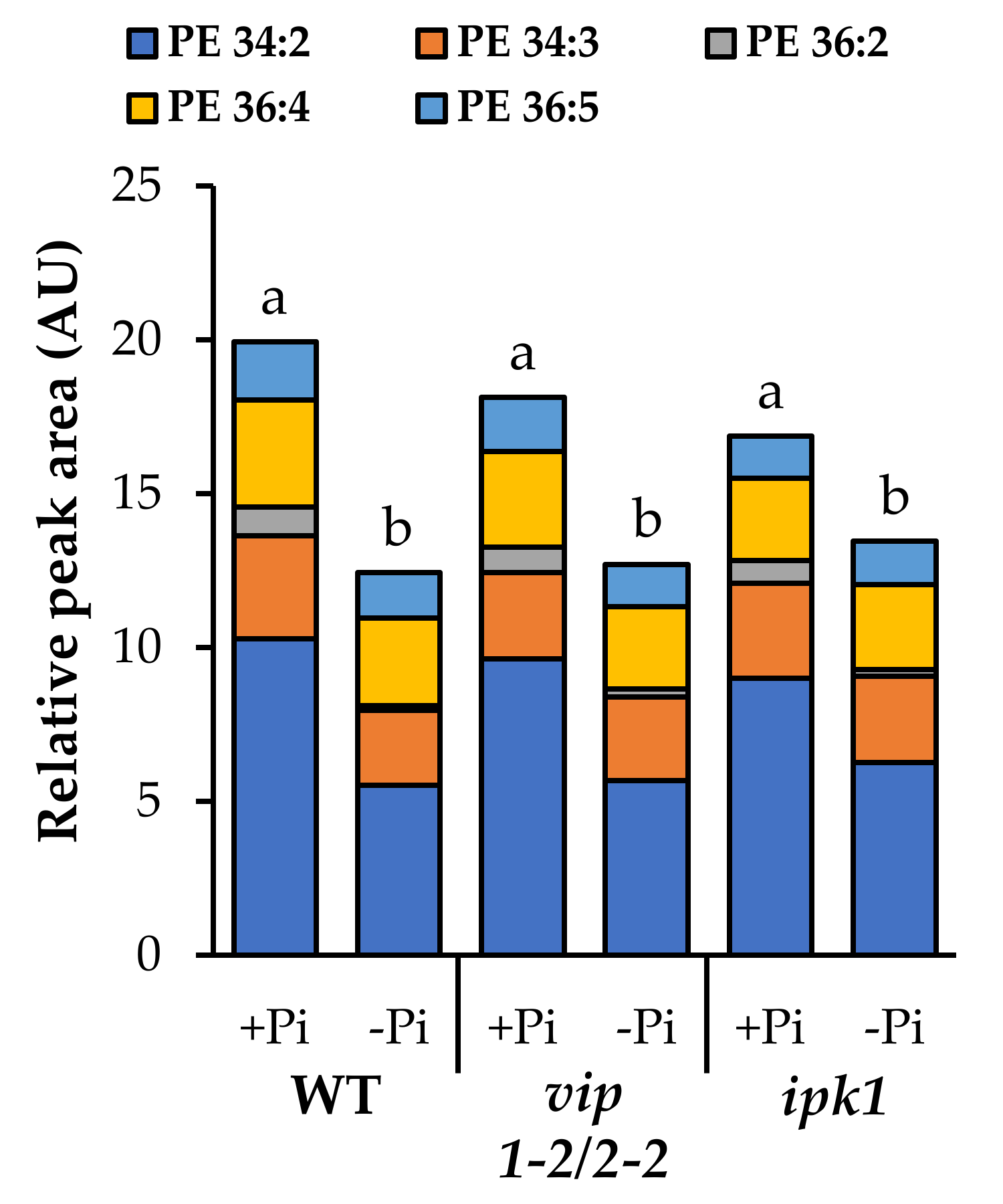

Supplement: Supplementary file 1 [file metabolites-11-00601-s001.zip › FigureS5A.tiff]

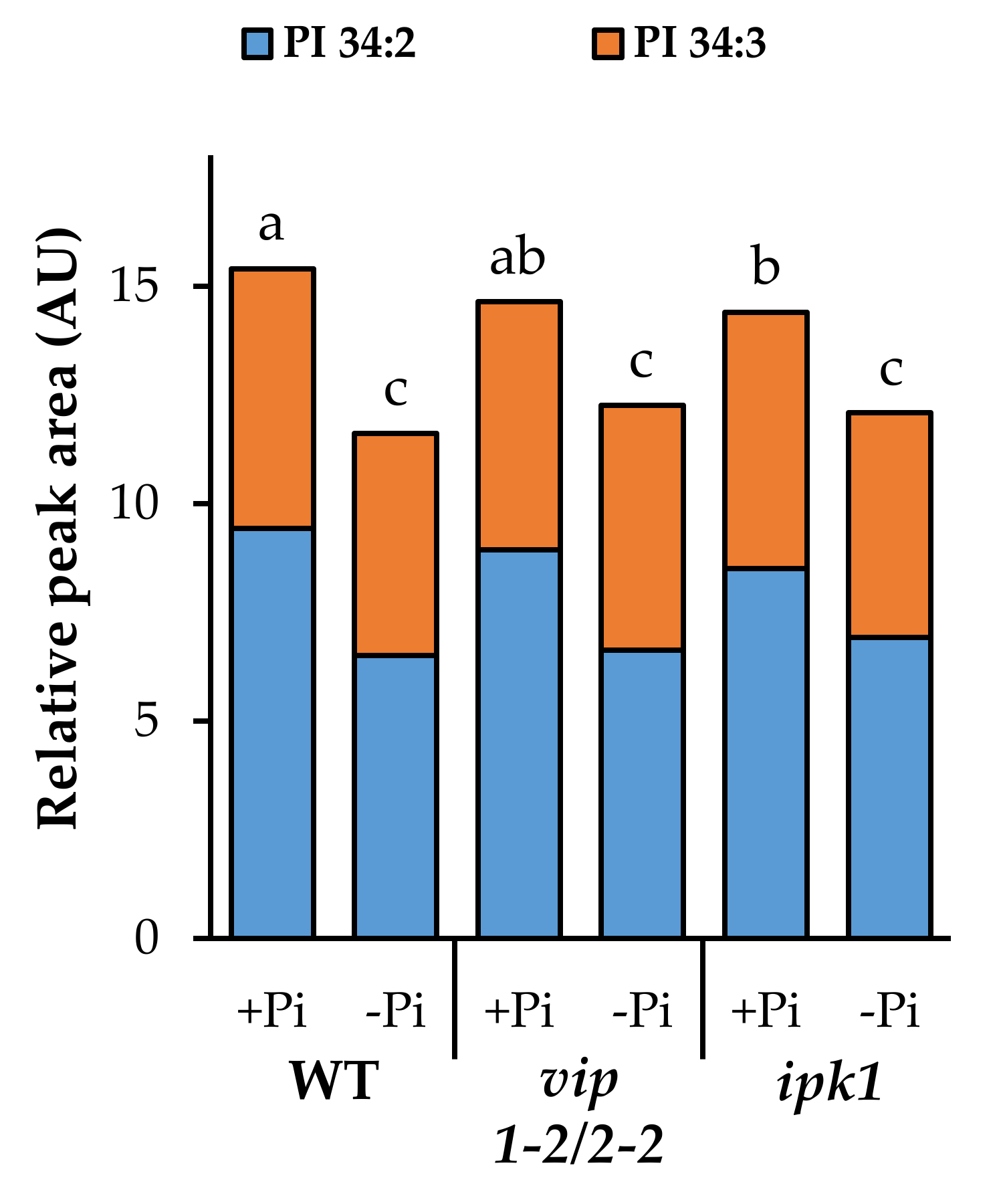

Supplement: Supplementary file 1 [file metabolites-11-00601-s001.zip › FigureS5B.tiff]

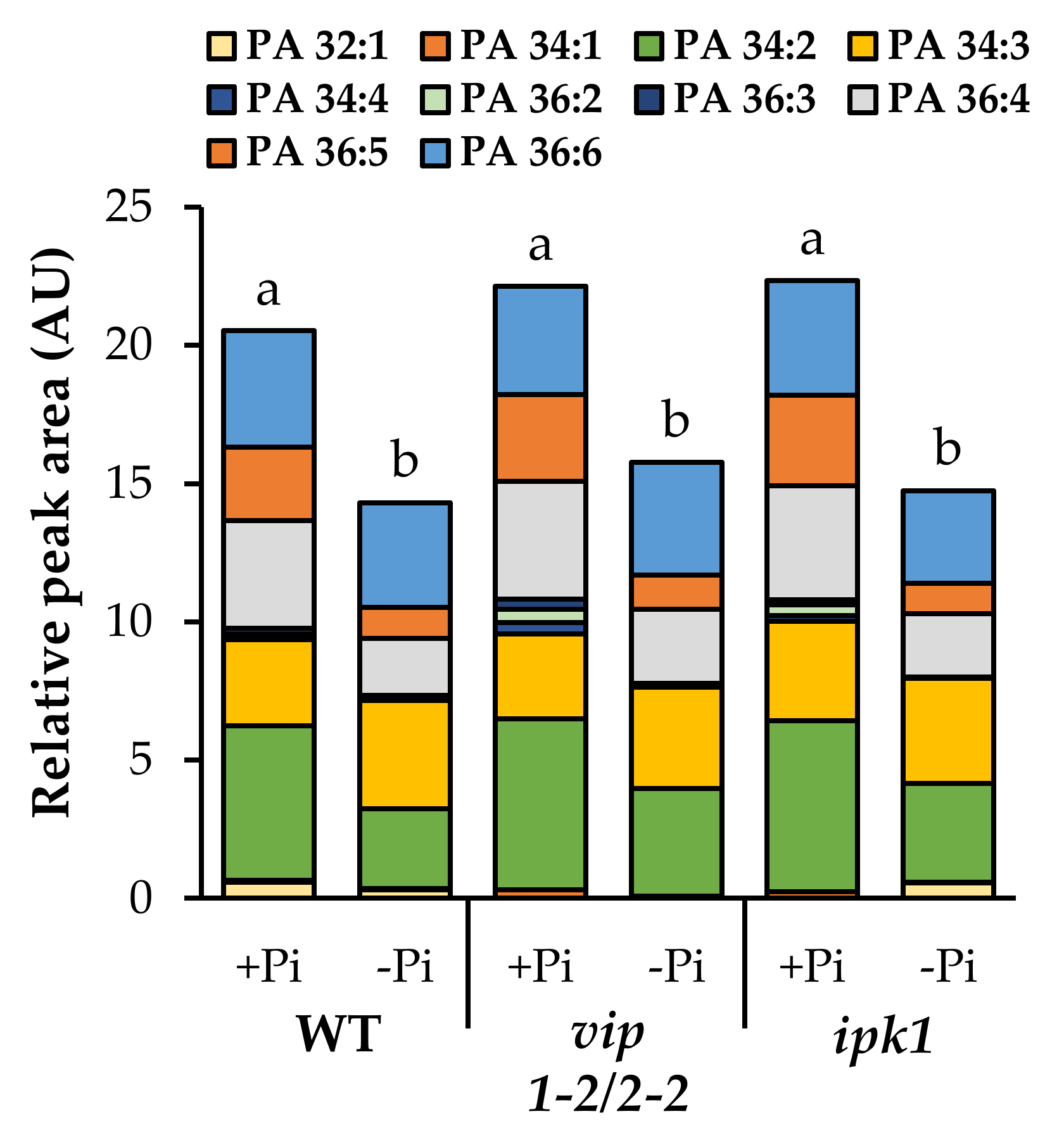

Supplement: Supplementary file 1 [file metabolites-11-00601-s001.zip › FigureS5C.tiff]

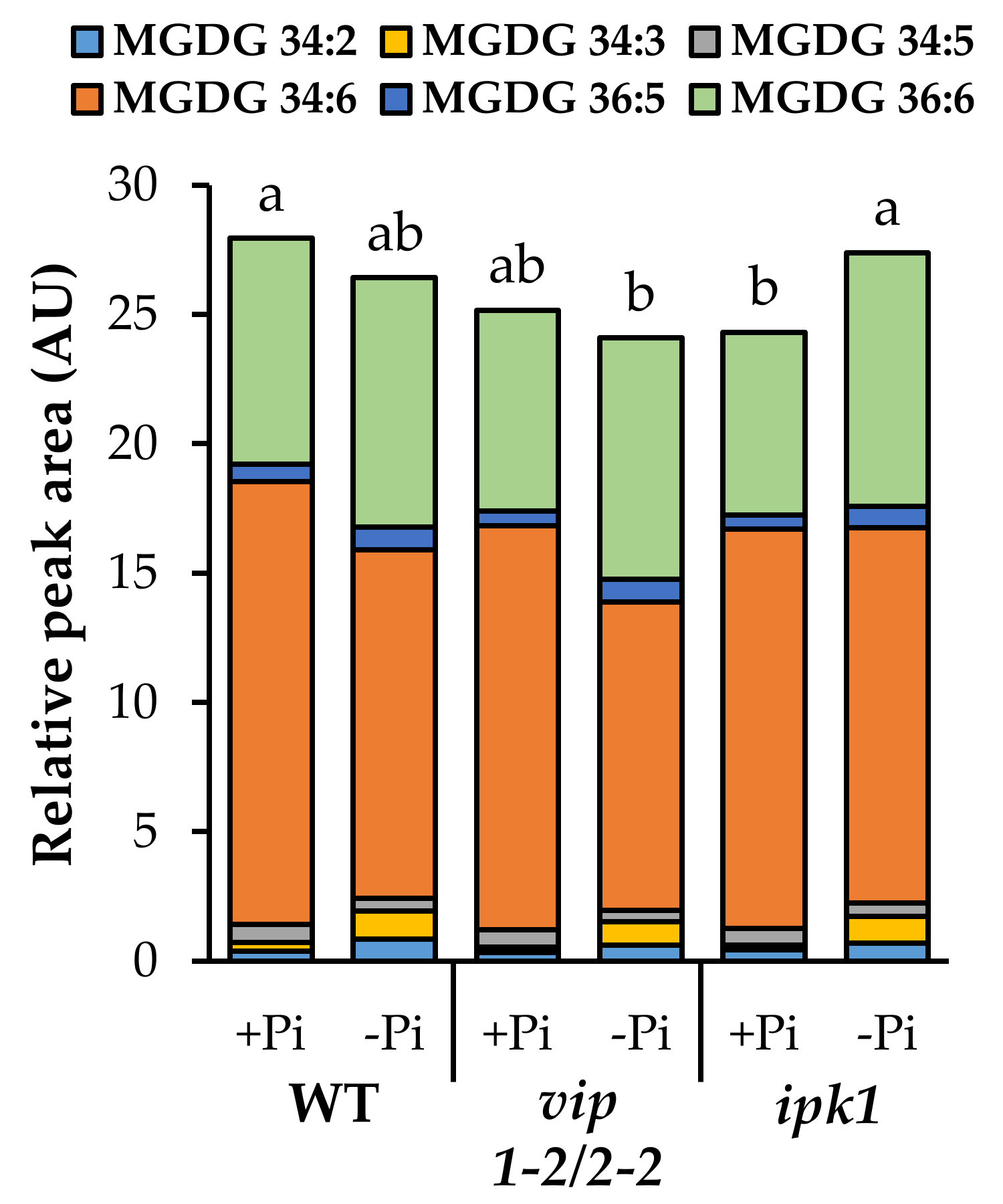

Supplement: Supplementary file 1 [file metabolites-11-00601-s001.zip › FigureS5D.tiff]

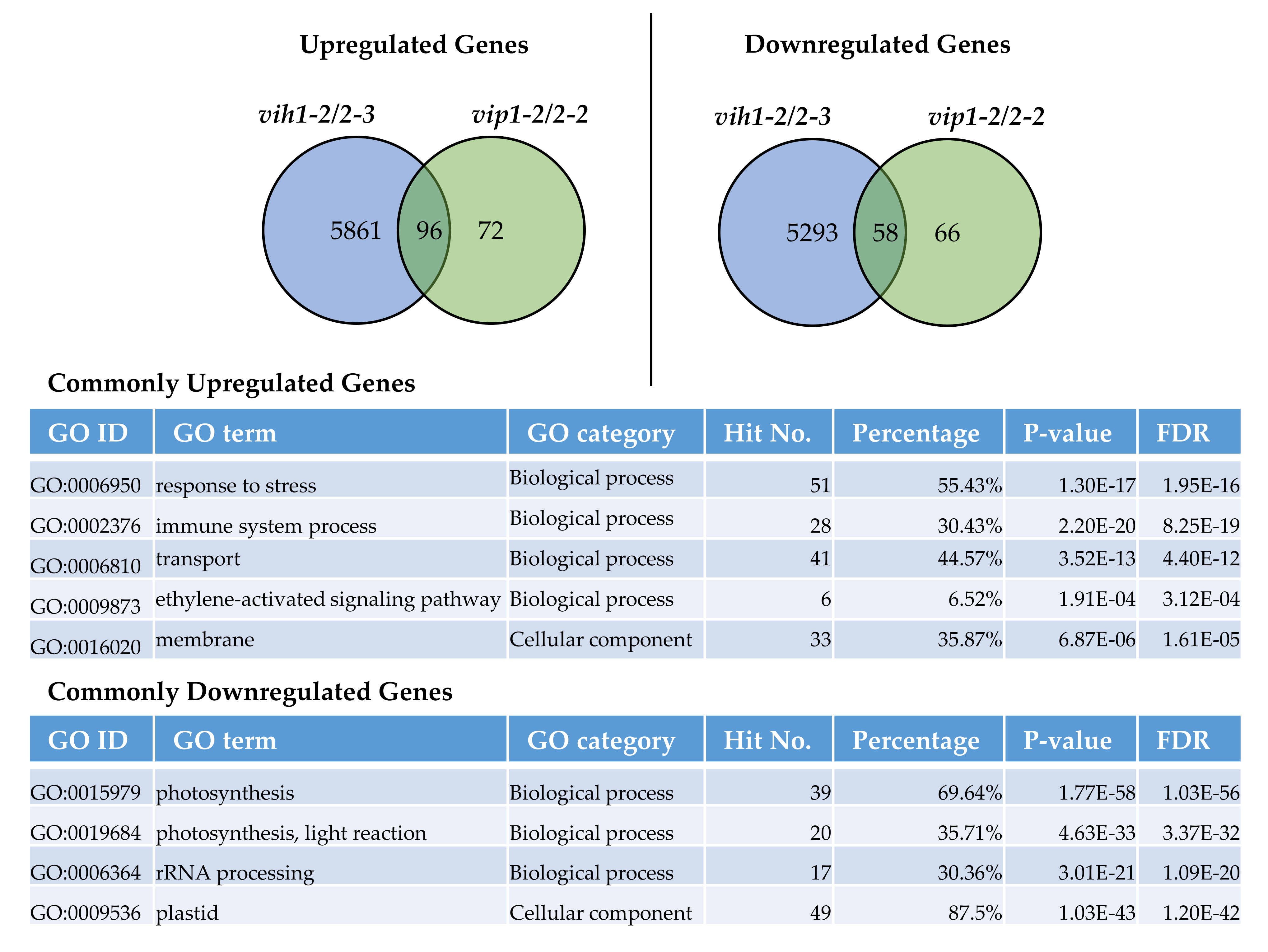

Supplement: Supplementary file 1 [file metabolites-11-00601-s001.zip › FigureS6.tiff]
